# Evidence that a STAT3 Mutation Causing Hyper IgE Syndrome Leads to Repression of Transcriptional Activity

**DOI:** 10.1155/2019/1869524

**Published:** 2019-10-13

**Authors:** Sameer Bahal, Maha E. Houssen, Ania Manson, Lorena Lorenzo, Mark A. Russell, Noel G. Morgan, Fariba Tahami, Sofia Grigoriadou

**Affiliations:** ^1^Department of Immunology, Royal London Hospital, Barts Health NHS Trust, London, UK; ^2^University of Exeter Medical School, UK; ^3^Biochemistry Department, Damanhour University, Egypt; ^4^Department of Immunology, Addenbrooke's Hospital, Cambridge, UK; ^5^Department of Immunology, Great Ormond Street Hospital, London, UK

## Abstract

We present the case of a 19-year-old female with a mild form of Autosomal Dominant Hyper IgE syndrome (HIES) associated with a loss-of-function mutation in *STAT3*. Within the first years of life she developed multiple, *Staphylococcus aureus* associated abscesses in the neck and face requiring frequent incision and drainage. Respiratory tract infections were not a feature of the clinical phenotype and a high resolution thoracic CT scan was unremarkable. Retained dentition was noted but fungal nail disease and recurrent thrush were absent. The total IgE was 970 IU/L, Lymphocyte counts and immunoglobulin levels were normal (IgG borderline 18.5 gr/L). There was suboptimal response to test immunisation with Pneumovax II vaccine. Th17 cell phenotyping revealed low levels of IL-17 expressing cells (0.3% of total CD4 T Cells numbers). Genetic analysis identified a missense mutation, N567D, in a conserved region of the linker domain of STAT3. Functional studies in HEK293 cells reveal that this mutation potently inhibits STAT3 activity when compared to the wildtype protein. This is consistent with other reported mutations in *STAT3* associated with HIES. However, surprisingly, the magnitude of inhibition was similar to another STAT3 mutation (V637M) which causes a much more severe form of the disease.

## 1. Introduction

Hyper IgE syndrome (HIES) is a rare primary immune deficiency and is characterised by elevated circulating levels of IgE. Patients typically experience eczema, lung, and skin infections, but other co-morbidities have also been described including brain and cardiac abnormalities. The autosomal dominant form of HIES is most commonly associated with inactivating mutations in STAT3 although HIES-associated mutations in DOCK8 and Tyk2 are reported [1].

The transcription factor STAT3 is a multifunctional protein, whose activity is controlled by a plethora of cytokines and growth factors acting at their cognate cell surface receptors. Activated STAT3 translocates to the nucleus where it binds to consensus sequences in the DNA to regulate target gene expression. A variety of mutations in STAT3 have been implicated in disease and, in addition to loss-of-function mutations associated with HIES [2], various activating mutations have also been described which may predispose to certain forms of cancer [3], autoimmune forms of neonatal diabetes, and various immune deficiencies [4], including CVID [5]. In the current report, we performed sequencing of samples from a patient with a mild form of HIES, to identify a missense mutation in the linker domain of STAT3 which caused a reduction in transcriptional activity and is likely to be causative for disease.

## 2. Patient Description

We present the case of a 19-year-old female with Autosomal Dominant HIES. She was born at 36 weeks gestation and early in life she developed multiple, *Staphylococcus aureus* associated abscesses in the neck and face requiring frequent incision and drainage. Respiratory tract infections were not a feature of the clinical phenotype and a thoracic CT scan was unremarkable. Retained dentition and mild eczema were noted but fungal nail disease and recurrent thrush were absent. The circulating total IgE was markedly elevated (970 IU/L, NR: 0-81 IU/L); T and B cell counts were normal but IgG was raised (18.5 gr/L). Complement C3 and C4 levels, and complement function tests were normal. There was a suboptimal response to test immunisation with Pneumovax II vaccine. The patient is currently managed with flucloxacillin 500 mg BD for *Staphylococcus aureus* prophylaxis. The disease activity calculated via the score previously described by Grimbacher et al., was 36 [6]. This is classed as indicating an indeterminate risk of HIES and reflects the mild/moderate phenotype [7].

## 3. Materials and Methods

### 3.1. Sanger Sequencing

Genomic DNA was isolated from whole blood. Coding genomic sequences and cDNA of STAT3 were purified with the QIAquick PCR purification kit (Qiagen, Hilden, Germany). Subsequently, PCR products were sequenced using the ABI PRISM BigDye Terminator cycle ready reaction kit V3.1 (Applied Biosystems). The sequencing was performed on a 3130xl Applied Biosystems Genetic Analyzer. Data analysis was performed with DNA Sequencing Analysis software, v5.2 (Applied Biosystems) and Sequencher v4.8 (Gene Codes Corp, Ann Arbor, Mich).

### 3.2. Cell Culture

A DMEM base medium supplemented with 10% foetal calf serum, 2 mM L-glutamine, 100 *μ*g/ml streptomycin, and 100 U/ml penicillin was used to culture HEK293 cells. Cells were sub-cultured upon reaching approximately 80% confluency.

### 3.3. Mutagenesis

Mutations within the human *STAT3* gene (Source Bioscience, Nottingham, UK) were introduced using the QuikChange site-directed mutagenesis kit (Agilent Technologies, CA, USA). The custom primers used to generate STAT3 variants were N567D; Fd ACAAGGTCAATGATATCGTCCAGCCAGACCCAG Rv: TCTGGGTCTGGCTGGACGATATCATTGACCTTGTG Y640F; Fd: AGTCCGTGGAACCATTCACAAAGCAGCAGCTG Rv: AGCTGCTGCTTTGTGAATGGTTCCACGGACTG V637M; Fd; AGACCCAGATCCAGTCCATGGAACCATACACAAAG Rv; TGCTTTGTGTATGGTTCCATGGACTGGATCTGGGTC. The success of mutagenesis was confirmed by full sequencing of inserts (Source Bioscience). The STAT3 inserts were digested out of a pENTR221 vector using *Psi*I to produce a blunt-ended product. This was treated with a calf intestinal phosphatase enzyme prior to insertion into a pcDNA5/FRT/TO expression vector at the *Eco*RV restriction site within the polylinker.

### 3.4. Reporter Assay

HEK293 cells were seeded into 24-well plates and transfected with 200 ng of the STAT3-reponsive dual luciferase Cignal reporter construct (Qiagen) and 400 ng of the various STAT3 constructs. Attractene transfection reagent (Qiagen) was used to facilitate DNA uptake, and STAT3 activity was assessed 24 h after transfection using a dual luciferase reporter assay system (Promega, Madison, WI, USA) and with luminescence detected using a Pherastar FS (BMG Labtech, Ortenberg, Germany). In some experiments, cells were treated with human IL-6 (R&D systems, Minneapolis, MN, USA) 4 h after transfection.

### 3.5. Western Blotting

Cells were lysed and whole cell protein was extracted [8]. Protein was denatured, loaded evenly onto a 4%–12.5% Bis-Tris polyacrylamide gel and separated by electrophoresis. Protein was then transferred onto a PVDF membrane prior to immunoblotting using an iBind Flex according to the manufacturer's guidelines (Thermo Fisher Scientific). Membranes were probed with STAT3 antisera (1 : 1000; Cell Signalling, Beverly, MA, USA) or beta-actin (1 : 2000; Sigma-Aldrich, Poole, Dorset, UK), and then with appropriate alkaline phosphatase conjugated secondary antibodies. CDP Star chemiluminescent reagent (Sigma-Aldrich) was used to detect bands, and this was visualised using a cDigit blot scanner.

### 3.6. Th17 Phenotyping

PBMCs were isolated from a 10 ml heparin blood sample using LymphoprepTM (Axis-Shield). The PBMCs were re-suspended in 4 ml of RPMI with 10% foetal bovine serum. Cells were either untreated or stimulated with 1 *µ*g/mL Staphylococcus enterotoxin B (SEB; Sigma) for 2 h. GolgiStop (BD Biosciences) was then added and cells were incubated for a further 16 h. Subsequently, cells were surface stained with CD3 (V500), CD4 (V450), and CD45RO (PerCP-Cy5.5) and intracellular stained with IL-4 (PE-Cy7), IFN-*γ* (Alexa Fluor®488), and IL-17A (Alexa Fluor®647). Intracellular staining was performed by using the BD Cytofix/Cytoperm™ Fixation/Permeabilization Kit (BD Biosciences). All antibodies were from BD Biosciences. Cells were analysed using an 8 colour BD FACSCanto.

## 4. Results

### 4.1. Th17 Profiling of Patient

HIES patients typically display reduced numbers of Th17 T-helper cells [9]. Th17 cell phenotyping revealed that Th17 cells comprised 0.3% of the total CD4+ T-cell number. This was below the normal range of >0.4%, and consistent with the diagnosis of Hyper IgE syndrome.

### 4.2. Functional Investigations

**Figure 1:**
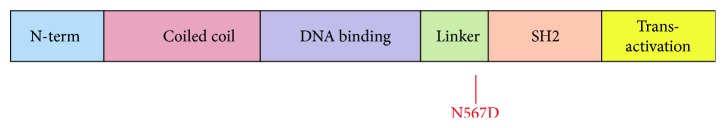
Schematic diagram of STAT3 protein structure showing the position of the N567D mutation identified from a patient with Hyper IgE syndrome.

Sanger sequencing of the patient's DNA revealed a missense mutation in the STAT3 gene. The variant was heterozygous with a nucleotide exchange (A to G) at position 1699 in exon 19, leading to an aspartate for asparagine substitution at position 567 within the linker domain (N567D) (Figure 1). The amino acid sequence is highly conserved at this region across multiple species [10].

**Figure 2:**
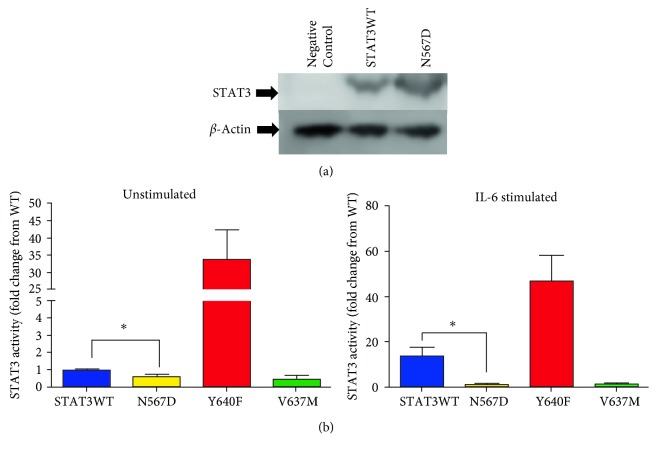
Transfection of N567D-STAT3 into cells increased STAT3 activity. (a) HEK293 cells were transfected with equal amounts of an empty vector, wildtype STAT3 or N567D and expression of STAT3 was studied by Western blotting. Results are representative of two independent experiments. (b) Cells were alternatively transfected with wildtype, N567D, and mutations which are known to activate (Y640F), and inactivate (V637M) STAT3. These cells were either grown in the presence or absence of 20 ng/ml IL-6 for 18 h, and transcriptional activity was determined using a dual-luciferase reporter assay (*n* = 3 − 6). ^*∗*^*P* < 0.05.

The N567D variant of STAT3 was generated by site-directed mutagenesis, and upon transfection into HEK293 cells, Western blotting analysis revealed an increase in STAT3 expression compared to cells transfected with empty vector (Figure 2(a)). Importantly, transfection of either the wildtype (WT) or mutant form of STAT3 resulted in approximately equal levels of protein expression (Figure 2(a)).

To study the impact of N567D on STAT3 transcriptional activity, a STAT3-responsive dual-luciferase reporter construct was employed, and this revealed that STAT3-N567D significantly inhibited protein activity compared to WT STAT3 (fold change from WT; N567D: 0.6 ± 0.2, *p* < 0.05) (Figure 2(b)). IL-6 is an agonist of STAT3 signalling, and treatment of HEK293 cells with 20 ng/ml IL-6 enhanced STAT3 activity by ~14-fold in cells expressing WT STAT3. Under IL-6 stimulated conditions the inhibitory effects of N567D were more pronounced, and STAT3 activity was reduced to levels comparable to those seen in unstimulated cells expressing WT STAT3 (Figure 2(b)). It is important to emphasise that, in these studies, expression of the mutant form was achieved in the context of the continued expression of the endogenous (wild type) STAT3 present in HEK293 cells. As such, the data are likely to be reflective of the situation occurring in vivo when a dominant heterozygous mutations is found.

To validate the assay system, previously reported STAT3 mutations which enhance (Y640F) or reduce (V637M) STAT3 activity were tested in parallel with N567D. STAT3 activity was modified in the expected direction under both basal and IL-6 stimulated conditions upon the transfection of cells with these variants (Figures 2(a) and 2(b)).

## 5. Discussion

Autosomal dominant HIES is caused by loss-of-function mutations in STAT3 [11]. Consistent with this, we describe a *de novo* mutation in the linker domain of STAT3 from a patient with a mild/moderate form of HIES that robustly inhibited the activity of STAT3 under both basal and IL-6 stimulated conditions. This mutation has been described in a single previous case with a more severe phenotype, although the authors did not examine its transcriptional activity *in vitro *[10]. Interestingly, despite the mild/moderate phenotype seen in our patient, the magnitude of STAT3 inhibition caused by the present mutation was comparable to that observed for other HIES associated mutants (e.g., V637M) which are reported to yield a much more severe disease phenotype [12]. This suggests that the precise location of the mutation within the structure of STAT3 may be critical for final determination of the disease phenotype even when the net outcome is a modest inhibition of transcriptional activity.

In considering the effects of the present mutation, it is known that STAT3 is crucial for the differentiation of T-cells to a Th17 phenotype [13], and in accord once with this circulating Th17 T-cell numbers were reduced in both patients in whom the mutation was found [10]. In addition, both patients had elevated IgE levels and recurrent infections with *Staphylococcus aureus*. However, some differences in phenotype were noted. For example, unlike our patient, the earlier case exhibited an allergic history, bone fractures as well as oral candidiasis.

The majority of STAT3 mutations were associated with HIES cluster in the SH2 domain, the DNA-binding domain and the transactivation domain of the protein [14]. To our knowledge, only two additional variants have been identified in the linker domain of STAT3, K531E [15], and I568F [16]. All STAT family members possess an *α*-helical linker between their SH2 and transactivation domains. Since this linker acts as a ridged spacer between functionally distinct groups, its disruption may result in altered protein function. In this context, targeted mutation of several conserved residues within the linker domain of STAT3 strongly reduced IL-6 stimulated transcriptional activity [17]. Similar data have been reported for STAT1, which shares high sequence homology with that of STAT3 in the linker region [18]. Merli and colleagues performed *in silico* investigations, examining the intramolecular interactions that occur with the N567 residue, mutated in the present variant [10]. This revealed that N567 interacts with K615, a highly conserved residue in the DNA binding domain, and that the aspartic acid substitution is likely to disrupt this interaction and impinge on DNA binding and dimer stability. In support of this, MUpro software, which calculates protein stability, predicts that N567D will decrease stability (ΔΔ*G* = −1.05) [19]. Taken together, these data support the notion that disruption of the linker domain (as in N567D) will impact STAT3 protein function.

The loss of STAT3 activity is an important driver of HIES, and therapeutic strategies to prevent or reverse this would be of interest to the field. In this context, a recent study revealed that certain small molecule compounds can improve the stability and activity of specific HIES-associated STAT3 mutants [20]. Whilst these findings have potentially exciting implications, they are based on *in vitro* studies and these molecules were only effective against the mutations which destabilised STAT3, presumably due to their upregulation of chaperone proteins. Thus, alternative methods to improve the activity of STAT3 are needed. Perhaps, small molecules targeted to the linker region of STAT3 may be one mechanism to achieve this.
